# Orbital Apex Malignant Lymphoma Diagnosed Using Whole-Body Computed Tomography

**DOI:** 10.7759/cureus.81191

**Published:** 2025-03-25

**Authors:** Taichi Higashida, Sotaro Mori, Kimitaka Katanazaka, Keiji Kurata, Risa Ashizaki, Takeshi Tanaka, Makoto Nakamura

**Affiliations:** 1 Division of Ophthalmology, Department of Surgery, Kobe University Graduate School of Medicine, Kobe, JPN; 2 Division of Neurology, Kobe University Graduate School of Medicine, Kobe, JPN; 3 Division of Medical Oncology and Hematology, Department of Medicine, Kobe University Graduate School of Medicine, Kobe, JPN; 4 Division of Gastroenterology, Department of Internal Medicine, Kobe University Graduate School of Medicine, Kobe, JPN

**Keywords:** diffuse large b-cell lymphoma (dlbcl), mri, neuro-ophthalmology, orbital apex lymphoma, whole-body ct

## Abstract

This article aims to report a case of orbital apex malignant lymphoma diagnosed using whole-body CT, emphasizing the challenges in diagnosis and the importance of timely investigation and treatment. A 69-year-old female with a history of rheumatoid arthritis and hypertension presented with a one-week history of headache and newly developed left eye ptosis. Initial examination revealed 20/20 visual acuity, left eye ptosis, and impaired upward eye movement. A subsequent orbital MRI, performed one week later, identified a mass extending from the left superior rectus muscle to the orbital apex. Elevated soluble IL-2 receptor levels were noted, and whole-body CT revealed multiple liver and pancreatic masses, as well as cardiac lesions. Biopsies from these sites led to a diagnosis of diffuse large B-cell lymphoma. Despite prompt initiation of chemotherapy, the optic nerve damage persisted, resulting in significant visual impairment. Whole-body CT imaging plays a crucial role in diagnosing challenging cases of orbital apex lesions by identifying primary or metastatic sites suitable for biopsy. This case emphasizes the necessity of prompt and comprehensive diagnostic evaluations and timely treatment initiation in suspected malignant lymphoma to prevent irreversible complications such as optic nerve damage.

## Introduction

Malignant lymphoma presents diagnostic challenges due to its propensity for lesions in diverse anatomical sites and a wide array of presenting symptoms. Within ophthalmology, intraocular malignant lymphoma manifesting as uveitis symptoms and mucosa-associated lymphoid tissue (MALT) lymphoma affecting periocular regions such as the conjunctiva and lacrimal gland are encountered sporadically [1]. While biopsies of the vitreous, conjunctiva, and lacrimal gland are typically diagnostic, lesions may infrequently arise within the intraconal space. In such cases, patients with preserved visual function may be reluctant to undergo biopsy because of the potential risk of visual impairment. In this report, we describe a case wherein malignant lymphoma involving the orbital apex was suspected, with the patient presenting with intact visual function upon initial evaluation. Subsequent whole-body CT revealed multiple masses in the liver and pancreas, along with cardiac lesions. Biopsies from these masses confirmed the diagnosis of diffuse large B-cell lymphoma (DLBCL).

## Case presentation

**Figure 1:**
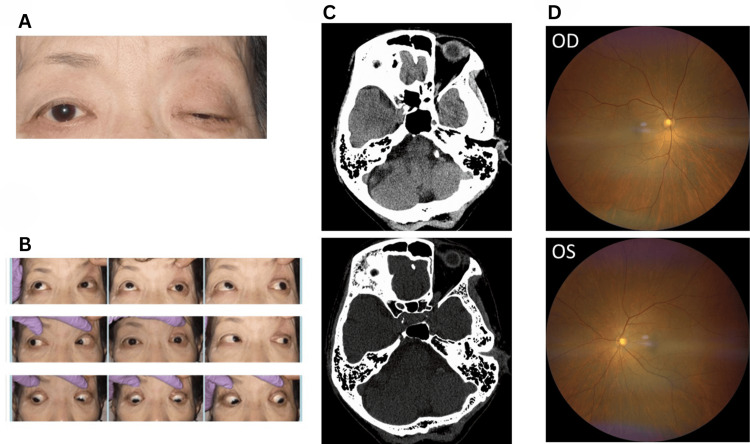
Ophthalmic examination findings and CT scanning images at initial visit (A) Photograph of the eyes and (B) eye movement assessment at the initial evaluation. The patient exhibited left eye ptosis and restricted upward movement of the left eye. (C) CT scan images showing no aneurysm in the internal carotid artery-posterior communicating artery region. (D) Fundoscopic examination of both eyes revealed no significant abnormalities. CT: computed tomography, OD: oculus dexter (right eye), OS: oculus sinister (left eye)

A 69-year-old female with a medical history of rheumatoid arthritis and hypertension presented with a one-week history of headache and left eye ptosis, which had developed the previous day. Upon arrival at our hospital, her corrected visual acuity was 20/20, accompanied by ptosis of the left eye and impaired upward left eye movement disorder (Figure 1A-1B). Examination revealed no abnormalities in pupillary movement or differences in pupil diameter between the left and right eyes, leading to a diagnosis of superior divisional oculomotor nerve palsy. Given the presence of headache, CT angiography of the head was conducted to rule out an aneurysm in the internal carotid artery-posterior communicating artery region. The absence of an aneurysm was confirmed by CT angiography, and the patient was scheduled for an orbital MRI at a later date. This CT scan did not reveal any bone destruction. Initial CT findings failed to detect any mass lesion within the left orbit (later revealed by MRI) (Figure 1C).

**Figure 2:**
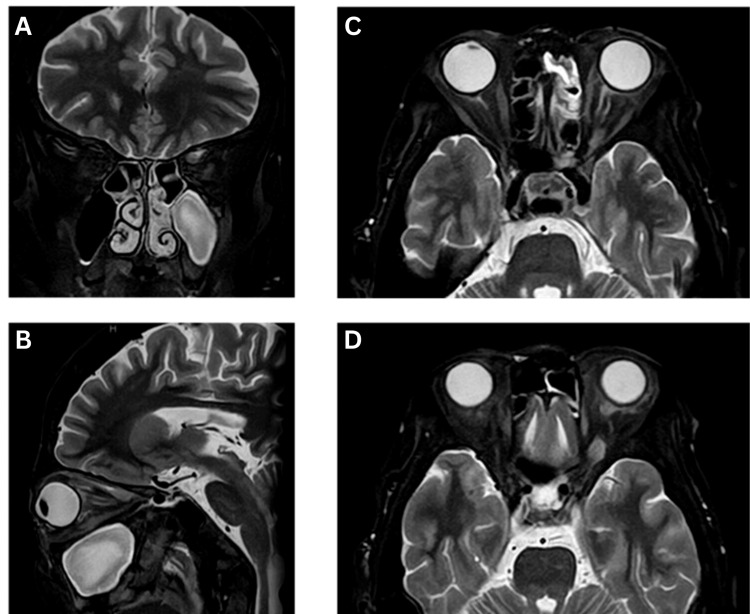
Orbital MRI images before treatment (A) Coronal section, (B) sagittal section, and (C-D) horizontal section. An irregular lesion was found in the muscle belly of the left superior rectus muscle. MRI: magnetic resonance imaging

One week later, orbital MRI unveiled a mass lesion extending from the muscle belly of the left superior rectus muscle to the orbital apex (Figure 2). The lesion was clearly delineated from the surrounding tissue in these images and appeared consistent with a benign tumor. However, it did not exhibit the typical imaging characteristics of cavernous hemangioma, pleomorphic adenoma, or optic nerve glioma, which are commonly observed in this region. Therefore, it was difficult to determine the tumor type based on imaging findings alone. Additionally, although no evidence of clear invasion beyond the orbit was observed, the possibility could not be completely excluded, making interpretation of the images challenging. A blood test on the first day of the patient's presentation showed an elevated soluble IL-2 receptor level (963.5 U/ml; normal range: 156.6-474.5 U/ml) (Table 1). Given the patient's history of rheumatoid arthritis, which was well controlled with methotrexate and adalimumab, the initially deemed unrelated elevated soluble IL-2 receptor level was reevaluated, suggesting a potential association with the orbital lesion. The presence of a suspicious lesion within the orbital muscle conus on MRI raised concerns for malignant lymphoma. Due to the potential risk of vision impairment associated with biopsy, a neurologist was consulted for cerebrospinal fluid analysis.

**Table 1:** Blood and cerebrospinal fluid test results before treatment PR3-ANCA: proteinase 3 anti-neutrophil cytoplasmic antibody, MPO-ANCA: myeloperoxidase anti-neutrophil cytoplasmic antibody, IgG4: immunoglobulin G4, T-SPOT: T-cell spot test, Na: sodium, K: potassium, Cl: chloride, Ca: calcium, IgG: immunoglobulin

Blood test results		
Complete blood count	Test value	Normal range
White blood cell (/µl)	5,800	3,300-8,600
Red blood cell (×10^6/μl)	3.71	3.86-4.92
Hemoglobin (g/dl)	12.4	11.6-14.8
Hematocrit (%)	36.9	35.1-44.4
Platelet (×10^3/μl)	184	158-348
Tumor-associated test		
Soluble interleukin 2 receptor (U/ml)	963.5	156.6-474.5
Immunologic test		
PR3-ANCA (U/ml)	<1	<3.5
MPO-ANCA (U/ml)	<1	<3.5
IgG4 (U/ml)	<6	11-121
Infection-associated test		
Rapid plasma reagin (R.U.)	<1.0	<1.0
Treponemal antibody	(-)	
β-D-Glucan (pg/mL)	<6	<11
T-SPOT	(-)	-
Endocrine test		
Thyroid-stimulating hormone (mIU/l)	2.22	0.61-4.23
Free T3 (pg/ml)	2.2	2.3-4.0
Free T4 (ng/dl)	1.19	0.9-1.7
Thyroglobulin antibody (IU/ml)	14	<28
Thyroid peroxidase antibodies(IU/ml)	<9	<16
Thyrotropin receptor antibody (IU/l)	<0.8	<2.0
Angiotensin-converting enzyme (IU/l)	18	7.7-29.4
Acetylcholine receptor antibody (nmol/l)	<0.3	<0.3
Biochemistry		
Na (mmol/l)	137	138-145
K (mmol/l)	4	3.6-4.8
Cl (mmol/l)	103	101-108
Ca (mg/dl)	10.1	8.8-10.1
Blood urea nitrogen (mg/dl)	11.7	8.0-20.0
Creatinine (mg/dl)	0.74	0.46-0.79
Erythrocyte sedimentation rate (mm/h)	33	<15
C-reactive protein (mg/dl)	0.07	<0.14
Aspartate aminotransferase (U/l)	26	13-30
Alanine aminotransferase (U/l)	15	7-23
Gamma-glutamyl transpeptidase (U/l)	24	9-32
Alkaline phosphatase (U/l)	75	38-113
Lactate dehydrogenase (U/l)	335	124-222
Creatine kinase (U/l)	55	41-153
Cholinesterase (U/l)	259	201-421
Uric acid (mg/dl)	11.7	8.0-20.0
Total protein (g/dl)	7	6.6-8.1
Albumin (g/dl)	4	4.1-5.1
Total bilirubin (mg/dl)	0.8	0.4-1.5
Total cholesterol (mg/dl)	171	146-219
Glucose (mg/dl)	78	73-109
Cerebrospinal fluid test results		
Cell counts (/µl)	1	<5
Protein (mg/dl)	50	15-45
Glucose (mg/dl)	42	73-109
Lactate dehydrogenase (U/l)	22	-
β2-Microglobulin (µg/l)	1821	-
Adenosine deaminase	<2.0	-
IgG index	0.5	<0.73
Soluble interleukin 2 receptor (U/ml)	<30	-
Angiotensin-converting enzyme (IU/l)	<1.0	-

**Figure 3:**
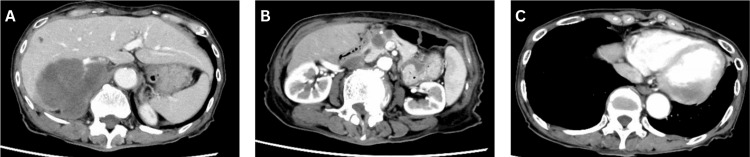
Whole-body CT images of the liver, pancreas, and cardiac sections Contrast-enhanced CT showed multiple hypovascular lesions in the liver (A), pancreas (B) and heart (C). CT: computed tomography

**Figure 4:**
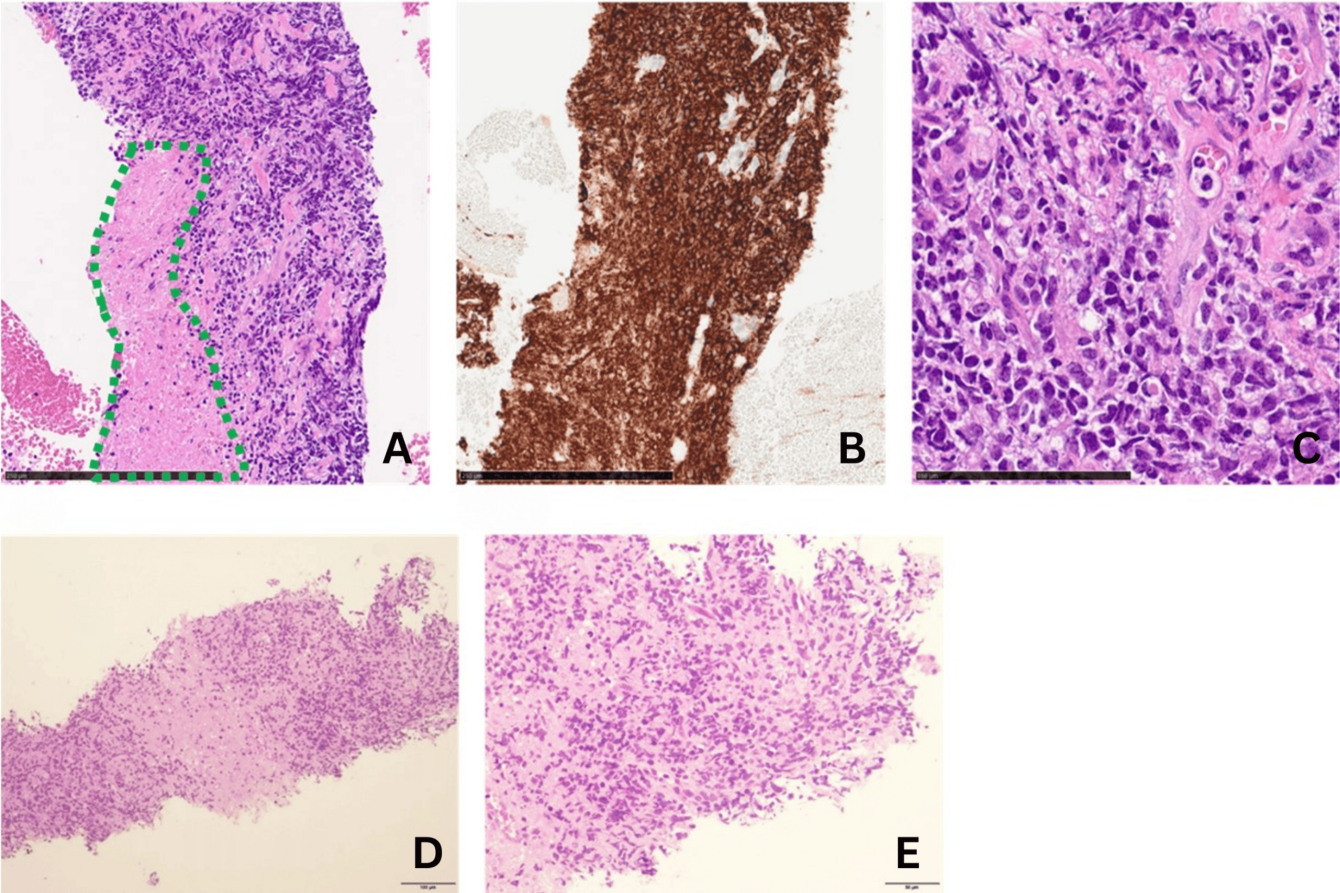
Microphotograph of liver and pancreas sections The sections were stained with hematoxylin-eosin (A, C, D, E) and CD20 (B). (A, B) Low-magnification image of the pancreas. The scale bar indicates 250 µm. Green areas indicate necrotic tissue (A). Atypical lymphocytes are diffusely positive for CD20 (B). (C) High-magnification image of the pancreas. The scale bar indicates 100 µm. Proliferation of atypical lymphocytes was observed. (D, E) Proliferation of lymphoma cells in the liver section. The scale bar indicates 100 µm (D) and 50 µm (E).

Subsequent cerebrospinal fluid examination revealed no significant abnormalities (Table 1), while whole-body CT disclosed multiple masses in the liver and pancreas, along with cardiac lesions (Figure 3). The patient was admitted to the gastroenterology department for biopsy, and tissue samples were obtained from the liver and pancreatic masses, leading to the diagnosis of DLBCL (Figure 4). By this time, one month had elapsed since the initial presentation, during which the patient's left eye had lost light reflex, resulting in complete loss of light perception and the development of optic nerve damage, culminating in orbital apex syndrome.

**Figure 5:**
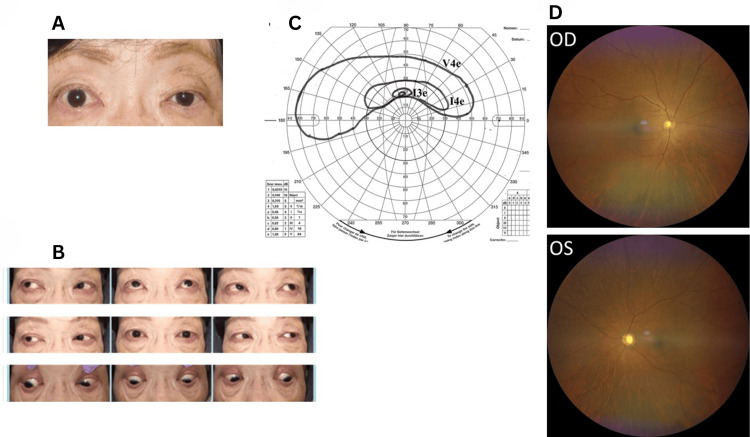
Ophthalmic examination findings and MRI scanning images after treatment Six months after treatment, her ptosis and ocular motility disorders had improved (A-B). However, the left optic nerve was atrophied, and visual field defects persisted (C-D). MRI: magnetic resonance imaging, OD: oculus dexter (right eye), OS: oculus sinister (left eye)

An oncologist was consulted for treatment. The patient underwent chemotherapy comprising six cycles of polatuzumab vedotin with rituximab, cyclophosphamide, doxorubicin, and prednisone (Pola-R-CHP), along with three courses of intrathecal methotrexate-cytarabine-dexamethasone (IT-MAD). Following completion of the four-month regimen, remission of oculomotor nerve palsy symptoms was observed, with resolution of ptosis and eye movement disorders (Figure 5A-5B). However, optic nerve damage persisted, leading to optic nerve atrophy, and the visual acuity remained unimproved at a corrected visual acuity of 20/1000 (Figure 5C-5D).

## Discussion

Orbital lymphoma represents 6-8% of ophthalmic tumors [2], with the majority, 57%, presenting as MALT lymphoma, while follicular lymphomas, DLBCL, and mantle cell lymphomas are also observed [3]. For intraorbital lesions, ophthalmologists, neurosurgeons, and otolaryngologists have proposed various surgical approaches, such as lateral orbitotomy, supraorbital, and transnasal methods. However, these approaches may present cosmetic and functional concerns due to craniotomy involvement, limited surgeon's field of view, and potential optic nerve damage. Consequently, observation is often considered for painless or non-visually impaired lesions [4]. In this case, as the patient's visual acuity remained intact during the initial examination, the possibility of developing optic neuropathy could not be determined at the initial examination.

Prognosis in malignant lymphoma varies depending on its subtype. For instance, DLBCL, as seen in this case, is categorized as aggressive and generally carries a poorer prognosis compared to indolent lymphomas such as follicular lymphoma [5]. Given the intraconal location of the lesion, rather than the conjunctiva or lacrimal gland [6], the prognosis was potentially unfavorable. Hence, a prompt diagnosis was deemed crucial for this case, and through whole-body CT scanning, an extraneous lesion suitable for biopsy was identified.

While performing whole-body CT scans for diseases of unknown cause is generally discouraged [7], this is particularly applicable to ophthalmologists who typically do not treat systemic diseases. However, when considering tumor metastasis, where the utility of whole-body CT and MRI has been noted, particularly in lymphoma cases, it becomes relevant [8]. In cases of undefined paralysis, consideration of paraneoplastic syndromes as differential diagnoses is warranted. Utilizing whole-body CT scans to identify primary and metastatic tumor sites can aid in diagnosis [9]. Moreover, reports [10] suggesting methotrexate as a risk factor for malignant lymphoma prompted suspicion of methotrexate-induced lymphoma in this case. Although there was speculation that discontinuing methotrexate alone could improve the condition [11], optic nerve damage had already occurred without light perception. Thus, expedited chemotherapy was deemed necessary.

Discovery of lesions outside the orbital region using whole-body CT scanning was pivotal in this case. Had these examinations proven ineffective, detecting the primary lesion would have been challenging. To further explore potential lesions, PET-CT, known for its heightened sensitivity compared to CT, was planned [12]. If no lesion had been found, performing a biopsy at the stage of optic nerve damage would have been considered, despite the risk of further optic nerve impairment.

## Conclusions

In cases involving mass lesions at the orbital apex, which pose challenges for biopsy, whole-body CT imaging with consideration for tumor metastasis aids in identifying primary or metastatic lesions, facilitating the attainment of a definitive diagnosis. In this instance, chemotherapy was initiated approximately one month after disease onset, resulting in the reduction of the orbital lesion. However, despite treatment, the optic nerve damage persisted. Thus, prompt investigation and treatment are imperative in cases of suspected malignant lymphoma.
